# Evaluating the clinical effectiveness of new beta-lactam/beta-lactamase inhibitor combination antibiotics: A systematic literature review and meta-analysis

**DOI:** 10.1017/ash.2021.217

**Published:** 2021-11-25

**Authors:** Geneva M. Wilson, Margaret A. Fitzpatrick, Kyle Walding, Beverly Gonzalez, Marin L. Schweizer, Katie J. Suda, Charlesnika T. Evans

**Affiliations:** 1 Center of Innovation for Complex Chronic Healthcare (CINCCH), Edward Hines Jr Veterans’ Affairs (VA) Hospital, Hines, Illinois; 2 Loyola University Chicago Stritch School of Medicine, Maywood, Illinois; 3 Carver College of Medicine, University of Iowa, Iowa City, Iowa; 4 Center for Access and Delivery Research and Evaluation (CADRE), Iowa City VA Health Care System, Iowa City, Iowa; 5 Center for Health Equity Research and Promotion, VA Pittsburgh Heath Care System, Pittsburgh, Pennsylvania; 6 Department of Medicine, University of Pittsburgh School of Medicine, Pittsburgh, Pennsylvania; 7 Department of Preventive Medicine, Center for Health Services and Outcomes Research, Northwestern University Feinberg School of Medicine, Chicago, Illinois

## Abstract

**Background:**

Ceftazidime/avibactam (C/A), ceftolozane/tazobactam (C/T), imipenem/relebactam (I/R), and meropenem/vaborbactam (M/V) combine either a cephalosporin (C/T and C/A) or a carbapenem antibiotic (M/V and I/R) with a β-lactamase inhibitor. They are used to treat carbapenem-resistant Enterobacterales (CRE) and/or multidrug-resistant *Pseudomonas aeruginosa* (MDRPA).

**Objective:**

We compared the pooled clinical success of these medications to older therapies.

**Methods:**

PubMed and EMBASE were searched from January 1, 2012, through September 2, 2020, for C/A, C/T, I/R, and M/V studies. The main outcome was clinical success, which was assessed using random-effects models. Stratified analyses were conducted for study drug, sample size, quality, infection source, study design, and multidrug-resistant gram-negative organism (MDRGNO) population. Microbiological success and 28- and 30-day mortality were assessed as secondary outcomes. Heterogeneity was determined using I^2^ values.

**Results:**

Overall, 25 articles met the inclusion criteria; 8 observational studies and 17 randomized control trials. We detected no difference in clinical success comparing new combination antibiotics with standard therapies for all included organisms (pooled OR, 1.21; 95% CI, 0.96–1.51). We detected a moderate level of heterogeneity among the included studies I^2^ = 56%. Studies that focused on patients with CRE or MDRPA infections demonstrated a strong association between treatment with new combination antibiotics and clinical success (pooled OR, 2.20; 95% CI, 1.60–3.57).

**Conclusions:**

C/T, C/A, I/R, and M/V are not inferior to standard therapies for treating various complicated infections, but they may have greater clinical success for treating MDRPA and CRE infections. More studies that evaluate the use of these antibiotics for drug-resistant infections are needed to determine their effectiveness.

Antibiotic-resistant infections are a serious healthcare concern in the United States; they cause an estimated 2.8 million infections and 35,000 death each year.^1^ Two of the most concerning organisms are carbapenem-resistant Enterobacterales (CRE) and multidrug-resistant *Pseudomonas aeruginosa* (MDRPA).^1^ CRE is classified as an urgent threat by the Centers for Disease Control and Prevention (CDC)^1^; it infects ∼13,000 people annually with an 8% mortality rate. MDRPA is defined as a serious threat by the CDC; it causes >32,000 infections each year, of which ∼2,700 are fatal.^1^ Common antibiotic treatments for these infections have historically involved the use of carbapenems, aminoglycosides, and colistin.^2,3^ However, the growing concern for antibiotic resistance, as well as treatment-limiting side effects, has led to the development of new combination antibiotics with either cephalosporins or carbapenems and a β-lactamase inhibitor.

Ceftolozane/tazobactam (C/T), is a combination fourth-generation cephalosporin and β-lactamase inhibitor that was approved for use by the FDA in 2014.^4^ C/T is primarily used for the treatment of MDRPA, but this combination can also be used to treat infections caused by extended-spectrum β-lactamase (ESBL)–producing organisms.^5,6^ Ceftazidime/avibactam (C/A), a combination third-generation cephalosporin and a novel β-lactamase inhibitor, was approved for use in 2015. C/A is primarily used for the treatment of CRE but is also used to treat infections caused by other multidrug-resistant gram-negative organisms (MDRGNOs).^5,7^ Meropenem/vaborbactam (M/V) and imipenem/relebactam (I/R) both combine a carbapenem with a novel β-lactamase inhibitor effective against *Klebsiella pneumoniae* carbapenemase (KPC)–producing Enterobacterales. They received FDA approval in 2018^8,9^ and 2019,^10^ respectively.

All of these new combination antibiotics are used to treat infections from several different sources including, but not limited to, complicated intra-abdominal and complicated urinary tract infections (c-IAI and c-UTI) and hospital- or ventilator-associated bacterial pneumonia (H/VABP).^3,11^ Clinical trials have individually shown that these medications are not inferior to standard therapies.^4,12,13^ However, a pooled analysis and comparison of the effectiveness of all these newer medications combined has not been conducted previously. Furthermore, the clinical trials that were conducted focused primarily on the treatment of infections from the same source and did not focus on the use of these drugs in patients with MDR infections. The goal of this study was to determine the effectiveness of these new combination antibiotics, with a particular focus on effectiveness in patients infected with CRE and MDRPA.

## Methods

### Article search

This systematic review was conducted using the Meta-analysis of Observational Studies in Epidemiology (MOOSE) criteria.^14^ PubMed and EMBASE were searched from January 1, 2012, through September 2, 2020, for studies that detailed the use of C/T, C/A, M/V, or I/R for the treatment of gram-negative infections. The following search terms were used to search both databases: “relebactam/tazobactam,” “ceftazidime/avibactam,” “imipenem/relebactam,” “meropenem/vaborbactam,” “cephalosporin/beta-lactamase inhibitor,” “*Pseudomonas aeruginosa*,” “ESBL organisms,” “multi-center study,” “beta-lactam,” “observational study,” “randomized control trial,” and “retrospective study.” The following study types were excluded: in vitro studies, non-English studies, animal studies, case studies, studies that did not evaluate either C/T, C/A, M/V or I/R, and studies that did not report a clinical success rate. Researchers G.W., K.W., and M.F. evaluated the studies for inclusion.

### Data abstraction and quality assessment

Researchers G.W., K.W., and M.F. all independently abstracted data from the included studies. The following information was collected from each article: patient demographics and medical comorbidities, infection characteristics, clinical and microbiological outcomes, adverse events, and mortality.

Observational studies were quality assessed using the Risk Of Bias for Non-randomized Studies of Intervention (ROBINS-I) tool^15^ developed by the Cochrane Collaboration. Randomized control trials (RCTs) were evaluated using the companion tool Revised Cochrane Risk-of-Bias tool for randomized trials (RoB-2).^16^ Studies were evaluated regarding the following domains: confounding, selection and randomization, intervention, missing data, outcomes, and reporting bias. Studies with a score of moderate risk of bias in 3 domains or high risk of bias in 1 domain were considered to have an overall moderate risk of bias. Those with a score of moderate in ≥4 domains or high in 2 or more domains were considered to have an overall high risk of bias. The confounding domain was not included on the RoB-2, so the RCTs were not scored on this topic.

### Outcome definitions

Clinical success was defined according to the definition provided by the study and was similarly defined across studies. Microbiological success was defined as a negative result from a culture that was taken from the site of infection at the conclusion of the antibiotic treatment for all infection sites except c-UTI. For c-UTI, microbiological success was defined as a bacterial concentration <10^4^ colony-forming units (CFU)/mL present in follow-up urine culture. Clinical success was evaluated by study type, sample size, quality, infection source, and study drug. A subanalysis of studies in which most of the study population had a multidrug-resistant organism (MDRO) infection was also performed.

### Statistical analyses

Pooled analysis was done using the Review Manager 5.3 program developed by the Cochrane Review group. Because of the variability in study design and intervention, random-effects models were generated using Mantel-Haenszel (M-H) weighting. Because consistent adjustments could not be made across all studies, unadjusted point estimates were pooled. The heterogeneity of each pooled comparison was assessed using an I^2^ value. The overall significance was determined by evaluating the *P* value for the pooled-effect estimate. For stratified analyses, significant differences between the groups were determined by comparing the pooled-effect estimate of each group via χ^2^ analysis. A funnel plot was created for the overall main analysis to determine whether publication bias existed among the included articles. All results were reported as pooled odds ratios (ORs) with 95% confidence intervals (CIs).

## Results

**Fig. 1.**
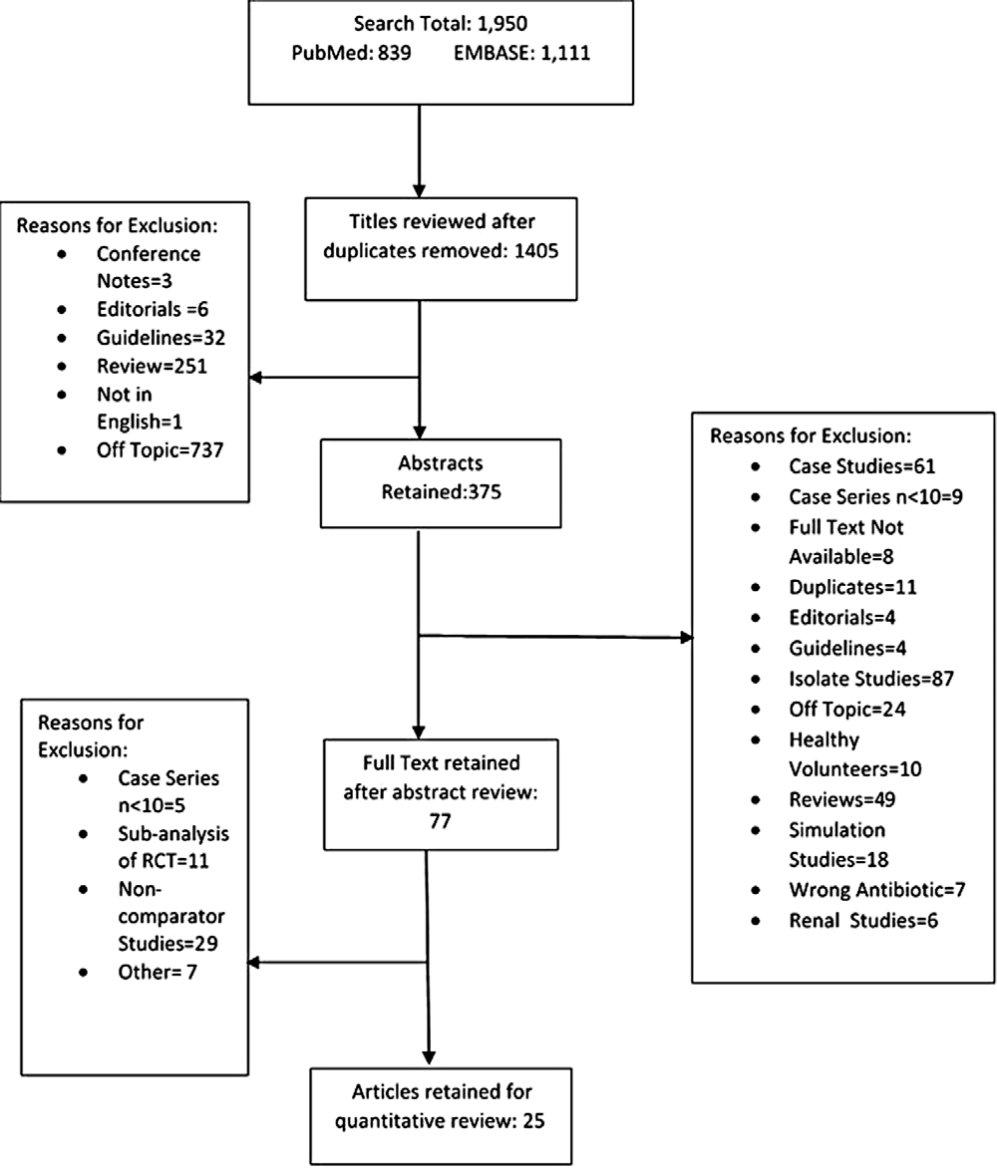
Search diagram for included studies.

In total, 1,950 articles were retrieved using our search terms: 839 from PubMed and 1,111 from EMBASE (Fig. 1). After applying the inclusion and exclusion criteria, 25 studies were retained: 17 randomized control trials^4,13,17–31^ and 8 observational studies.^12,32–38^ The duration of the randomized control trials was significantly shorter than the observational studies, with an average time of 23.0 months compared to 49.6 months (Table 1). C/A was the most evaluated antibiotic combination (11 studies), followed by C/T (7 studies), I/R (4 studies), and M/V (3 studies). The observational studies included were mostly based in the United States (5 of 8 studies) as opposed to the RCTs, which were all global with 1 exception. Carbapenems (primarily meropenem) were the most common comparison antibiotic (64% of studies), followed by colistin/polymyxins (32%) and aminoglycosides (20%). All of the observational studies primarily included patients with MDRO infections as opposed to the RCTs, in which infection source was emphasized over organism susceptibility. The primary organisms reported were *Klebsiella pneumonia*, *Escherichia coli*, *Enterobacter* spp, and *Pseudomonas aeruginosa*. Some studies did report the percentage of isolates that were nonsusceptible to the study drug, but because it was unclear whether these isolates met the criteria to be considered CRE or MDRPA, they were not included in the subanalyses. Patient comorbidities were not widely reported in the RCTs. Among the observational studies, the most frequently reported comorbidities across all studies were type 2 diabetes (29.6%), cancer (16.9%), and kidney disease (16.5%) (Table 2).

**Table 1.** Demographic Information for Included Studies

Author, Year	Study Design	Location	Sample Size	Study Drug	Comparison Drug	Duration, Months
Ackley, 2020	Retrospective cohort	USA	131	Meropenem-vaborbactam	Ceftazidime/avibactam	44
Bradley, 2019	RCT	Global	83	Ceftazidime-avibactam	Meropenem	22
Carmeli, 2016	RCT	Global	333	Ceftazidime-avibactam	Imipenem, meropenem	19
Caston, 2017	Retrospective cohort	Spain	31	Ceftazidime-avibactam	Aminoglycosides, carbapenems, and colistin	45
Fernandez-Cruz, 2019	Case-control	Spain	57	Ceftolozane-tazobactam	Piperacillin/tazobactam, meropenem, and colistin	23
Kaye, 2018	RCT	Global	550	Meropenem-vaborbactam	Piperacillin/tazobactam	17
Lucasti, 2013	RCT	Global	203	Ceftazidime-avibactam	Meropenem	9
Lucasti, 2014	RCT	Global	122	Ceftolozane-tazobactam	Meropenem	9
Lucasti, 2016	RCT	Global	351	Imipenem-relebactam	Imipenem	21
Mazuski, 2016	RCT	Global	1066	Ceftazidime-avibactam	Meropenem	26
Mills, 2019	Retrospective Cohort	USA	115	Ceftolozane-tazobactam	Not Reported	24
Motsch, 2019	RCT	Global	47	Imipenem-relebactam	Imipenem and colistin	23
Pogue, 2020	Retrospective Cohort	USA	200	Ceftolozane-tazobactam	Polymyxins and aminoglycosides	89
Qin, 2017	RCT	China, Korea, Vietnam	441	Ceftazidime-avibactam	Meropenem	6
Shields, 2017	Retrospective Cohort	USA	109	Ceftazidime-avibactam	Aminoglycosides, carbapenems, and colistin	97
Sims, 2017	RCT	Global	298	Imipenem-relebactam	Imipenem/cilastatin	30
Solomkin, 2015	RCT	Global	993	Ceftolozane-tazobactam	Meropenem	20
Titov, 2020	RCT	Global	537	Imipenem-relebactam	Piperacillin/tazobactam	39
Torres, 2019	RCT	Global	870	Ceftazidime-avibactam	Meropenem	33
van Duin, 2018	Prospective Cohort	USA	137	Ceftazidime-avibactam	Colistin	54
Vazquez, 2012	RCT	Global	137	Ceftazidime-avibactam	Imipenem/cilastatin	19
Vena, 2020	Case-control	Italy	48	Ceftolozane-tazobactam	Colistin and aminoglycosides	21
Wagenlehner, 2015	RCT	Global	1083	Ceftolozane-tazobactam	Levofloxacin	26
Wagenlehner, 2016	RCT	Global	1033	Ceftazidime-avibactam	Doripenem	22
Wunderink, 2018	RCT	Global	77	Meropenem-vaborbactam	Ceftazidime/avibactam, carbapenems, aminoglycosides, polymyxins	30

Note. RCT, randomized control trial.

**Table 2.** Clinical Features of Included Studies

Author, Year	Clinical Success Definition		Comorbidities			Infection Source		
		MDRPA/CRE %^a^	Diabetes,%	Kidney Disease, %	Cancer, %	c-IAI, %	c-UTI, %	H/VABP, %
Ackley, 2020^b^	Survival at 30 d and resolution of the signs and symptoms of infection	100^c^	47.3	32.1	21.4	14.8	14.5	37.4
Bradley, 2019	Resolution of signs and symptoms of infection or improvement such that no further treatment/intervention is needed	NR	NR	NR	NR	100	0.0	0.0
Carmeli, 2016	Resolution of signs and symptoms of infection or improvement such that no further treatment/intervention is needed	100^c,d^	NR	NR	NR	7	93	0.0
Caston, 2017^b^	Resolution of all signs and symptoms of infections at 14 d after onset of antibiotic treatment	78.8^d^	12.9	6.5	90.3	6.5	3.2	19.4
Fernandez-Cruz, 2019^b^	Not clearly reported	100^d^	7.0	5.3	100.0	0.0	21.1	24.6
Kaye, 2018	Complete resolution or improvement of signs and symptoms of infection	NR	48.7	17.6	NR	0.0	100	0.0
Lucasti, 2013	Resolution of signs and symptoms of infection or improvement such that no further treatment/intervention is needed	NR	NR	NR	NR	100	0.0	0.0
Lucasti, 2014	Resolution of signs and symptoms of infection or improvement such that no further treatment/intervention is needed	NR	NR	NR	NR	100	0.0	0.0
Lucasti, 2016	Resolution of signs and symptoms of infection or improvement such that no further treatment/intervention is needed	NR	NR	NR	NR	100	0.0	0.0
Mazuski, 2016	Resolution of signs and symptoms of infection or improvement such that no further treatment/intervention is needed	NR	8.1	NR	NR	100	0.0	0.0
Mills, 2019^b^	Clinical cure by 14 d of definitive therapy	100^d^	0.0	0.0	0.0	0.0	0.0	100.0
Motsch, 2019	Resolution of baseline signs and symptoms of infection	100^d^	NR	NR	NR	15	59	26
Pogue, 2020^b^	Resolution signs and symptoms of infection with the initial study regimen without therapy modification for failure or toxicity	100^d^	35.0	17.0	0.0	13.5	0.0	69.5
Qin, 2017	Resolution of signs and symptoms of infection or improvement such that no further treatment/intervention is needed	NR	9.8	NR	NR	100	0.0	0.0
Shields, 2017^b^	30-d survival and resolution of signs and symptoms of infection	100^c^	32.1	0.0	0.0	45.9	11.9	12.8
Sims, 2017	Determined by comparing baseline signs and symptoms with those after treatment	NR	NR	NR	NR	0.0	100	0.0
Solomkin, 2015	Complete resolution or significant improvement in signs and symptoms of index infection such that no further treatment/intervention is needed	NR	30.1	NR	NR	100	0.0	0.0
Titov, 2020	Resolution of baseline signs and symptoms plus no nonstudy antibiotics needed	NR	NR	NR	NR	0.0	0.0	100
Torres, 2019	Patient was alive and all signs and symptoms of pneumonia had resolved or improved such that no further treatment/intervention was needed	NR	26.7	NR	NR	0.0	0.0	100
van Duin, 2018^b^	Alive in hospital or discharged home	97.0^c^	43.8	32.1	13.1	0.0	13.9	21.9
Vazquez, 2012	Resolution of signs and symptoms of infection or improvement such that no further treatment/intervention is needed	NR	NR	NR	NR	0.0	100	0.0
Vena, 2020^b^	Clinical cure at 14 ds after start of treatment	100^d^	20.8	25.0	18.8	0.0	0.0	56.3
Wagenlehner, 2015	Reduction in severity of all baseline signs and symptoms and worsening of none	NR	72.9	10.3	NR	0.0	100	0.0
Wagenlehner, 2016	Resolution of UTI specific symptoms except flank pain from baseline to day 5 of treatment	NR	10.0	NR	NR	0.0	100	0.0
Wunderink, 2018	Resolution of signs and symptoms of infection such that no further treatment/intervention is needed	100^c^	NR	NR	NR	8.5	34.0	10.6

Note. c-IAI, complicated intra-abdominal infection; c-UTI, complicated urinary tract infection; NR, not reported.

a Percentage of study population with drug resistant infection.

b Observational study.

c Carbapenem-resistant Enterobacteriaceae.

d Multidrug-resistant *Pseudomonas aeruginosa*.

**Fig. 2.**
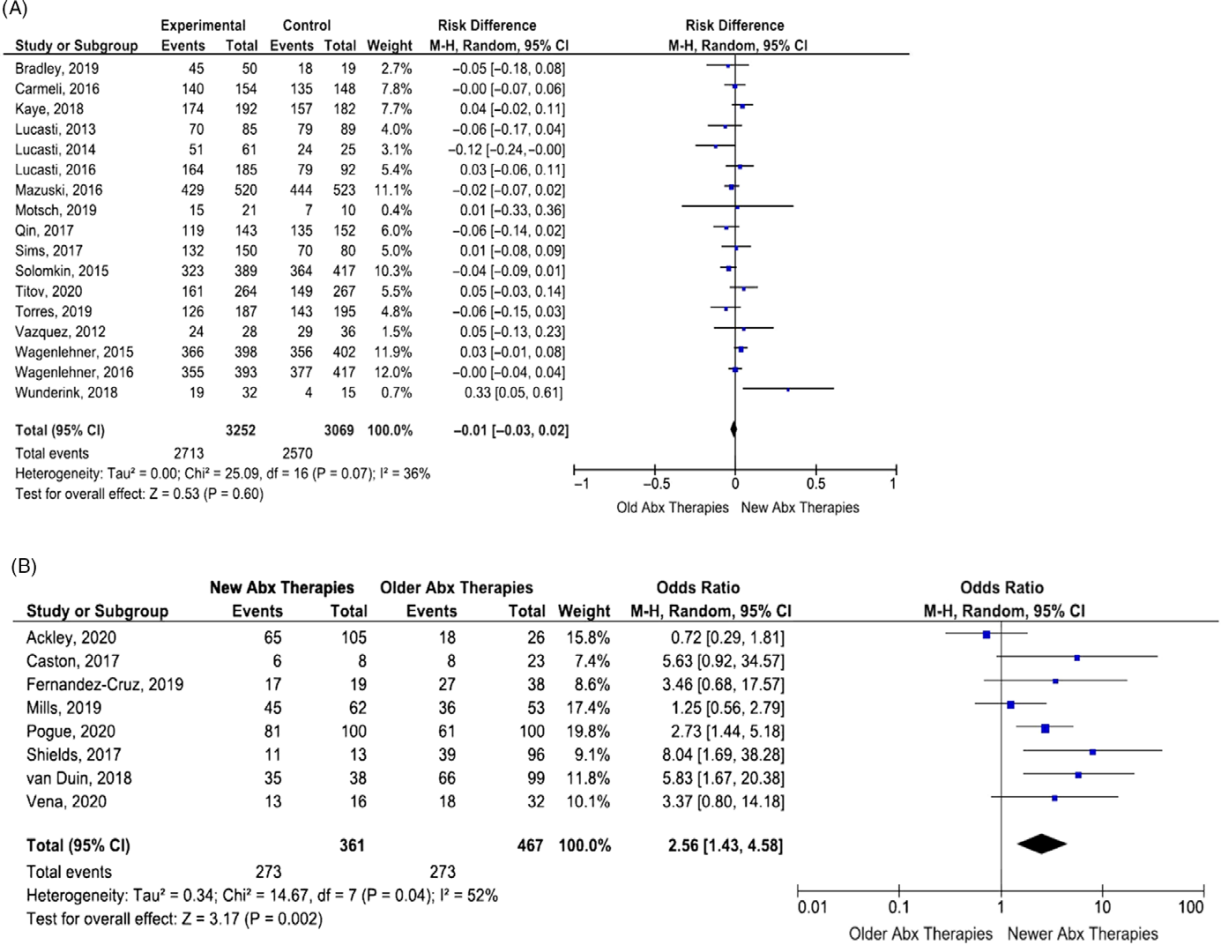
Pooled analysis of clinical success in all included studies. (A) Pooled analysis of all randomized control trials. (B) Pooled analysis of all observational studies.

**Fig. 3.**
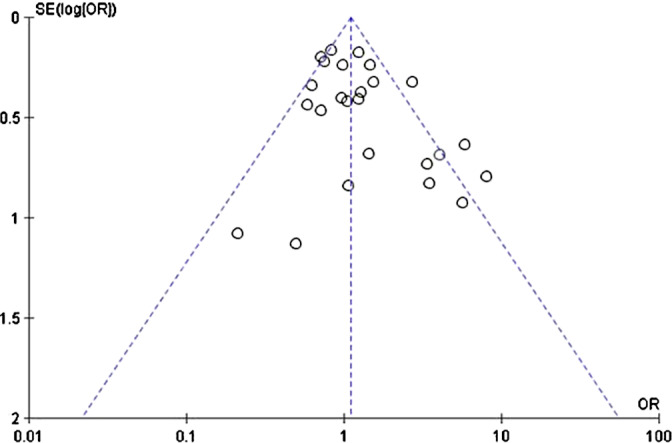
Funnel plot for all included studies.

The pooled effect of the new combination antibiotics was not inferior to older therapies for the main outcome of clinical success (pooled OR, 1.21; 95% CI, 0.96–1.51; *P* = .11). We detected a moderate level of heterogeneity among the included studies (I^2^ = 56%) (Fig. 2). The funnel plot did not show evidence of publication bias (Fig. 3). Also, 12 studies evaluated the secondary outcome of microbiological success. Among these studies, we detected increased odds of microbiologic success associated with the use of the new combination therapies (pooled OR, 1.27; 95% CI, 1.04–1.56; *P* = .02) (Table 3). We detected less heterogeneity in this comparison (I^2^ = 35%). When comparing results for the 8 observational studies to the 17 RCTs, the new combination antibiotics were associated with significantly greater odds of clinical success in the observational studies (pooled OR, 2.56; 95% CI, 1.43–4.58; *P* = .04), whereas we detected no significant association in the RCTs (pooled OR, 0.98; 95% CI, 0.82–1.17; *P* = .15).

**Table 3.** Stratified and Subanalyses of the Pooled Odds of Clinical Success

Outcomes	Included Studies	Pooled Odds Ratio (95% CI)	*P* Value	I^2^ Value, %
**Study type**				
Observational	8	2.56 (1.43–4.58)	.04	52
RCT	17	0.98 (0.82–1.17)	.15	27
**Bias**				
Low risk of bias	19	1.34 (1.03–1.61)	.03	66
Moderate risk of bias	6	0.95 (0.59–1.53)	.85	0
**Study size**				
<150 patients	12	1.96 (1.11–3.44)	.02	49
>150 patients	13	1.03 (0.84–1.27)	.76	50
**Predominant antibiotic**				
Ceftazidime/avibactam	11	1.07 (0.75–1.55)	.70	58
C/A RCTs	8	0.82 (0.67–1.00)	.05	0
Ceftolozane/tazobactam	7	1.46 (0.84–2.53)	.18	70
C/T RCT’s	3	0.92 (0.68–1.23)	.55	73
Imipenem/relebactam	4	1.21 (0.91–1.62)	.19	0.0
Meropenem/vaborbactam	3	1.48 (0.66–3.29)	.34	55
**MDRPA/CRE subanalysis**				
All MDRPA and CRE studies	11	2.20 (1.60–3.57)	.001	50
MDRPA only	6	2.22 (91.45–3.39)	.0002	0
CRE only	4	3.14 (0.93–10.57)	.06	74
RCTs	3	1.48 (0.61–3.63)	.39	40
Ceftazidime/avibactam	4	3.53 (1.06–11.73)	.04	71
Ceftolozane/tazobactam	4	2.21 (1.40–3.48)	.0006	0

Note. CI, confidence interval; RCT, randomized control trial. C/A, ceftazidime/avibactam; C/T, ceftolozane/tazobactam; MDRPA, multidrug-resistant *Pseudomonas aeruginosa*; CRE, carbapenem-resistant Enterobacterales.

Moreover, 11 studies (3 RCTs and 8 observational studies) primarily enrolled patients with either MDRPA or CRE. The pooled odds ratio for clinical success among the 3 MDRPA/CRE RCTs showed a stronger association compared to the overall RCT result (pooled OR, 1.48; 95% CI, 0.61–3.63; *P* = .39); however, it was not statistically significant. We detected moderate heterogeneity in this subanalysis (I^2^ = 40%), and 1 large RCT accounted for 49.7% of the comparison. Among these 11 studies, 6 studies enrolled patients with MDRPA, 4 studies included a CRE study population, and 1 study enrolled patients with both infections (Table 3). We detected a stronger association between the new combination antibiotic and clinical success in the MDRPA studies (pooled OR, 2.22; 95% CI, 1.45–3.39; *P* = .0002) compared to the CRE studies (pooled OR, 3.14; 95% CI, 0.93–10.57; *P* = .06). C/T and C/A were each evaluated in 4 studies, M/V was studied in 2 studies, and I/R was evaluated in 1 study. A stratified subanalysis of the 4 studies of C/A versus C/T revealed that both antibiotic combinations were strongly associated with clinical success: C/A pooled OR of 3.53 (95% CI, 1.06–11.73; *P* = .04) versus C/T pooled OR of 2.21 (95% CI, 1.40–3.48; *P* = .0006). We detected significantly more heterogeneity among the C/A studies than the C/T studies: I^2^ = 71% and 0%, respectively.

The studies included 3 main infection sources: hospital- or ventilator-associated pneumonia (H/VABP), complicated urinary tract infection (c-UTI), and complicated intra-abdominal infection (c-IAI). Studies in which the patient population consisted of a majority of any one of these infection types were included in a stratified analysis. New combination antibiotics were not associated with significantly increased odds of clinical success in H/VABP (pooled OR, 1.40; 95% CI, 0.86–2.26; *P* = .17); however, they were associated with increased odds of clinical success in c-UTI (pooled OR, 1.31; 95% CI, 1.01–1.71; *P* = .04). For c-IAIs, the new combination antibiotics were associated with a decreased odds of clinical success compared to the older therapies (pooled OR, 0.74; 95% CI, 0.55–0.99; *P* = .04). In this comparison, we detected an overall significant difference between the pooled effects of clinical success by infection source (*P* = .0008) as well as a large amount of heterogeneity (I^2^ = 79.2%).

A second stratified analysis of each antibiotic (C/A, C/T, I/R, and M/V) was also completed (Table 3). However, we detected no association between any 1 antibiotic and odds of clinical success. We also found no difference between the groups regarding this comparison (*P* = .79). According to the quality assessment, 19 studies had a low risk of bias compared to 6 with a moderate risk of bias. No studies were considered to have a high risk of bias (Table 4). We detected a stronger association among the studies with a low risk of bias compared to those with a moderate risk of bias: pooled OR of 1.34 (95% CI, 1.03–1.61; *P* = .03) versus pooled OR of 0.95 (95% CI, 0.59–1.53; *P* = .85). However, we detected significantly more heterogeneity among those studies with a low risk of bias compared to those with moderate risk: I^2^ = 66% versus 0%, respectively.

**Table 4.** Quality Assessment of Included Studies

Author, Year	Confounding Bias^a^	Selection/Randomization Bias^b^	Intervention Bias	Missing Data	Outcomes Bias	Reporting Bias	Overall Bias Score
Ackley, 2020	Low	Moderate	Low	Low	Low	Moderate	Low
Bradley, 2019	N/A	Low	Low	Low	Low	High	Moderate
Carmeli, 2016	N/A	Moderate	Moderate	Low	Low	Moderate	Moderate
Caston, 2017	Low	Low	Low	Low	Low	Moderate	Low
Fernandez-Cruz, 2019	Low	Moderate	Low	Low	Moderate	Moderate	Moderate
Kaye, 2018	N/A	Low	Low	Moderate	Low	Low	Low
Lucasti, 2013	N/A	Low	Low	High	Low	Low	Moderate
Lucasti, 2014	N/A	Low	Low	Low	Low	Low	Low
Lucasti, 2016	N/A	Low	Low	Low	Low	Moderate	Low
Mazuski, 2016	N/A	Low	Low	Low	Low	Moderate	Low
Mills, 2019	Low	Low	Low	Low	Moderate	Moderate	Low
Motsch, 2019	N/A	Moderate	Low	Moderate	Low	Moderate	Moderate
Pogue, 2020	Low	Low	Low	Low	Moderate	Moderate	Low
Qin, 2017	N/A	Low	Low	Low	Low	Moderate	Low
Shields, 2017	Moderate	Low	Low	Low	Moderate	Low	Low
Sims, 2017	N/A	Low	Low	Moderate	Low	Moderate	Low
Solomkin, 2015	N/A	Low	Low	Moderate	Low	Low	Low
Titov, 2020	N/A	Low	Low	Low	Low	Moderate	Low
Torres, 2019	N/A	Low	Low	Low	Low	Moderate	Low
van Duin, 2018	Low	Low	Low	Low	Moderate	Moderate	Low
Vazquez, 2012	N/A	Low	Low	High	Low	Moderate	Moderate
Vena, 2020	Low	Moderate	Low	Low	Moderate	Low	Low
Wagenlehner, 2015	N/A	Low	Low	Moderate	Low	Moderate	Low
Wagenlehner, 2016	N/A	Low	Low	Moderate	Low	Moderate	Low
Wunderink, 2018	N/A	Low	Low	Moderate	Low	Moderate	Low

Note. N/A, not applicable.

a Confound bias domain is not included in the RoB-2 for randomized control studies.

b Domain is labeled as selection bias in the ROBINS-I tool and randomization in the RoB-2.

To determine the effect of study sample size on association, a final stratified analysis was conducted comparing studies with cohorts of >150 patients versus those with <150 patients. We detected a stronger association among those studies with study cohorts of <150 (pooled OR, 1.96; 95% CI, 1.11–3.44; *P* = .02) compared to larger studies with cohorts of >150 (pooled OR, 1.03; 95% CI, 0.84–1.27; *P* = .72). The heterogeneity between these 2 groups was comparable: I^2^ = 49% versus 50% respectively. Among the 25 included studies, 9 measured 28- or 30-day mortality. We detected a protective association between the use of new combination antibiotics and mortality (pooled OR, 0.50; 95% CI, 0.33–0.75; *P* = .0007) and a low level of heterogeneity among these studies I^2^ = 23%.

## Discussion

In this meta-analysis of 25 studies that evaluated the clinical success of C/A, C/T, I/R, and M/V for the treatment of gram-negative infections, these therapies were not inferior to older standard therapies (pooled OR, 1.21; 95% CI, 0.96–1.51; *P* = 0.11). In patient populations with CRE and MDPRA infections, the new combination antibiotics proved superior to standard therapies (pooled OR, 2.20; 95% CI, 1.60–3.57; *P* = .001). These results agree with those of previous studies we have completed. A meta-analysis of 29 studies evaluating C/T, C/A, and M/V, the pooled clinical success rates for those antibiotics was 73.3% (95% CI, 68.9%–77.5%).^39^ However, that analysis did not include studies with a comparator group and therefore could not analyze the performance of the new combination antibiotics compared to older therapies.^39^ Additionally, the clinical effectiveness of I/R was not included in that analysis. A review of MDRGNO bloodstream infections found a decrease in 30-day mortality associated with C/A use.^40^ C/A and C/T were associated with an increased odds of clinical success among cancer patients with CRE and MDRPA infections.^41^

The 2020 Infectious Diseases Society of America (IDSA) guidelines for the antimicrobial treatment of gram-negative infections^42^ recommend C/A, I/R, and M/V as preferred treatments for CRE infections. The guidelines also list I/R, C/A, and C/T as preferred treatments for MDRPA infections. The results from our meta-analysis support the use of the new combination antibiotics for CRE and MDRPA infections over older treatments such as carbapenems, for which there is growing incidence of resistance, and polymyxins, for which toxicity can limit treatment.^42^

Although we detected no statistical association between the new combination antibiotics and clinical success, there was a significant association between the combination antibiotics and microbiological success (pooled OR, 1.27; 95% CI, 1.04–1.56; *P* = .02). The lack of evaluation for microbiological success in our included studies could be related to the difficulty of retrieving repeat cultures from some body sites (c-IAI and H/VABP). However, 50% of the studies that evaluated microbiologic success were studies evaluating c-UTI in which retrieving a repeat culture was a noninvasive procedure.

Interestingly, we detected a difference in the association between clinical success and infection type. We detected no association between clinical success with the newer antibiotics for H/VABP. However, the newer antibiotics were more effective against c-UTI, whereas the older therapies were more effective against c-IAI. These differences may be driven by infection-related factors. c-IAI infections are more difficult to treat because they are more dependent upon adequate source control and are often polymicrobial, requiring treatment with antimicrobials active against both gram-positive and gram-negative organisms.^17,23^ Further evaluation of these antibiotics for the treatment of c-IAI is needed to determine additional contributing factors to this association. Fewer included studies have focused on H/VABP infections; thus, more clinical trials focused on this infection type may yield a stronger association.

Although there is significant overlap between the types of infections treated by each of these new medications, there are some differences in the organisms targeted by each antibiotic. C/A, M/V, and I/R are recommended for the treatment of CRE infections, whereas C/A, C/T, and I/R are recommended for the treatment of MDRPA. To account for these differences in target organism, a stratified analysis of each study drug was conducted. However, the result showed no differences between the association of any 1 drug with patient outcomes. Finally, we detected a strong association between 28- and 30-day mortality and the use of the newer antibiotics. This association may be driven by studies that focused on the patient populations with MDRO infections because 7 of the 9 included studies in this subanalysis were of patients with resistant infections.

This meta-analysis offers an in-depth review of the new combination antibiotics approved for the treatment of complicated and drug-resistant infections. One strength of this article is the comparative analysis of the performance of these drugs in different patient populations (MDRO and infection source). Another is the evaluation of the results by different study designs. The inclusion of global studies is important because MDRGNO rates differ geographically.

This study also has several limitations. We did not include observational and MDRGNO studies for M/V and I/R. Because these antibiotics were recently approved, significantly fewer studies detailed their effectiveness. A future update may yield more publications focused on M/V or I/R. Additionally, the stronger association observed between the new combination antibiotics and clinical success among the observational studies maybe due to residual confounding in these studies that was not present in the RCTs. More RCTs evaluating these drugs in MDRGNO patient populations are needed to confirm this. RCTs may also have shown less significant clinical success rates due to enrollment of healthier patients who were more likely to survive infections regardless of antibiotic treatment. Lastly, we did not assess the adverse drug events associated with the newer versus older therapies, and this factor could be key. Even though efficacy was not inferior, the safety of the newer therapies could have been superior to older therapies.

In conclusion, this systematic review and meta-analysis showed that the use of new β-lactam/β-lactamase inhibitor combination antibiotics yielded comparable clinical success rates and better microbiologic success rates compared to older standard therapies across multiple infection types. Furthermore, the new combination antibiotics were associated with greater odds of clinical success in studies focused on MDRGNO infections, such as CRE and MDRPA. These results support the most recent IDSA guidelines that recommend these antibiotics as the preferred treatment option for CRE and MDRPA. However, these studies were primarily conducted on C/A and C/T, and more studies are needed to evaluate I/R and M/V in patients infected with MDRGNOs.
